# Embryo development and live birth resulted from artificial oocyte activation after microdissection testicular sperm extraction with ICSI in patients with non-obstructive azoospermia

**DOI:** 10.3389/fendo.2023.1123541

**Published:** 2023-02-21

**Authors:** Xi Zhang, Li Li, Wenhong Zhang, Yang Luo, Yuling Mao, Hongzi Du, Lei Li

**Affiliations:** ^1^ Department of Obstetrics and Gynecology, Center for Reproductive Medicine, Guangdong Provincial Key Laboratory of Major Obstetric Disease, The Third Affiliated Hospital of Guangzhou Medical University, Guangzhou, China; ^2^ Key Laboratory for Reproductive Medicine of Guangdong Province, The Third Affiliated Hospital of Guangzhou Medical University, Guangzhou, China

**Keywords:** intracytoplasmic sperm injection, microdissection testicular sperm extraction, artificial oocyte activation, non-obstructive azoospermia, fertility rate, live birth rate

## Abstract

**Introduction:**

The application of microdissection testicular sperm extraction (micro-TESE) to retrieve the sperm of patients with non-obstructive azoospermia (NOA) has greatly increased. Patients with NOA often have poor quality sperm. Unfortunately, there are few studies on artificial oocyte activation (AOA) performed on patients who successfully retrieved motile and immotile sperm by micro-TESE after intracytoplasmic sperm injection (ICSI). Therefore, this study sought to obtain more comprehensive evidence-based data and embryo development outcomes to aid consultation of patients with NOA who opted to receive assisted reproductive techniques and to determine whether AOA needs to be performed in different motile sperm after ICSI.

**Methods:**

This retrospective study involved 235 patients with NOA who underwent micro-TESE to retrieve adequate sperm for ICSI between January 2018 and December 2020. A total of 331 ICSI cycles were performed in the 235 couples. Embryological, clinical, and neonatal outcomes were demonstrated comprehensively between motile sperm and immotile sperm using AOA and non-AOA treatment.

**Results:**

Motile sperm injection with AOA (group 1) showed significantly higher fertility rate (72.77% *vs.* 67.59%, *p*=0.005), 2 pronucleus (2PN) fertility rate (64.33% *vs.* 60.22%, *p*=0.036), and miscarriage rate (17.65% *vs.* 2.44%, *p*=0.018) compared with motile sperm injection with non-AOA (group 2). Group 1 had comparable available embryo rate (41.29% *vs.* 40.74%, *p*=0.817), good embryo rate (13.44% *vs.* 15.44%, *p*=0.265), and without an embryo for transfer rate (10.85% *vs.* 9.90%, *p*=0.815) compared with group 2. Immotile sperm injection with AOA (group 3) displayed significantly higher fertility rate (78.56% *vs.* 67.59%, *p*=0.000), 2PN fertility rate (67.36% *vs.* 60.22%, *p*=0.001), without an embryo for transfer rate (23.76% *vs.* 9.90%, *p=*0.008), and miscarriage rate (20.00% *vs.* 2.44%, *p*=0.014), but significantly lower available embryo rate (26.63% *vs.*40.74%, *p*=0.000) and good embryo rate (15.44% *vs.* 6.99%, *p*=0.000) compared with group 2. In groups 1, 2, and 3, the rates of implantation (34.87%, 31.85% and 28.00%, respectively; *p*=0.408), clinical pregnancy (43.87%, 41.00%, and 34.48%, respectively; *p*=0.360) and live birth (36.13%, 40.00%, and 27.59%, respectively; *p*=0.194) were similar.

**Discussion:**

For those patients with NOA from whom adequate sperm were retrieved for ICSI, AOA could improve fertilization rate, but not embryo quality and live birth outcomes. For patients with NOA and only immotile sperm, AOA can help achieve acceptable fertilization rate and live birth outcomes. AOA is recommended for patients with NOA only when immotile sperm are injected.

## 1 Introduction

Non-obstructive azoospermia (NOA) is the most severe form of male infertility, characterized by the inability of the testes to produce mature sperm. NOA accounts for 60% of all patients with azoospermia (1). In 1999, Schlegel (2) reported that testicular sperm was obtained by cutting open the testes under magnification of a surgical microscope to search for the curving spermatogenic tubules, which was the first application of microdissection testicular sperm extraction (micro-TESE) in the field of assisted reproductive techniques. This procedure provided a new positive outlook for male patients with NOA.

Micro-TESE has gradually become a popular surgical technique with a high sperm retrieval rate and low tissue loss. Compared with men with normal spermatogenesis, patients with NOA had significantly lower rates of fertilization and pregnancy in testicular sperm undergoing an intracytoplasmic sperm injection (ICSI) cycle. In these patients the sperm retrieved through micro-TESE surgery had poor morphology and motility, increased oxidative stress levels, and higher degrees of DNA fragmentation. In addition, after cryopreservation, the reactive oxygen species significantly increased the DNA fragmentation of testicular sperm, resulting in chromatin damage, aneuploidy, mosaicism, and DNA damage, which contributes to poor embryo quality and increased miscarriage rates. Furthermore, the testicular sperm of men with impaired spermatogenesis may have a reduced rate of fertilization after ICSI due to lower sperm maturity relative to those of men with normal spermatogenesis, or fertilization may fail because the sperm cannot trigger oocyte activation (3, 4). In these cases, assisted oocyte activation (AOA) methods, including mechanical stimuli (5), electrical pulses (6), and chemical stimuli (7, 8) aim to induce artificial calcium (Ca^2+^) rises in the oocytes cytoplasm. Currently, Ca^2+^ ionophores, such as ionomycin and calcimycin, are most widely used in clinical application to keep intracellular Ca^2+^ levels sufficiently high (9). Over the past 20 years, AOA treatment with Ca^2+^ ionophores has been successfully used in infertile patients with failed or low fertilization (10, 11) and severe male factor infertility (12).

Studies have shown that AOA can improve fertilization rate in patients with ejaculatory sperm head malformation, at least one ICSI fertilization failure, and low fertilization rate (<30%) (13, 14). Nasr-Esfahani et al. (15) used AOA combined with ionemycin to improve the fertilization rate and ovulation rate of patients with teratospermia. Some reproductive centers have also found that AOA could improve the embryo quality of epididymal sperm ICSI in patients with OA when comparing the effect of epididymal sperm combined with testicular sperm in patients with OA and patients with NOA (16). Furthermore, many studies have focused on the relationship of defective sperm with low activation ability and oocyte activation. However, due to few centers being available for micro-TESE surgery and low sperm retrieved, how to select micro-TESE sperm for ICSI and whether AOA is required after injecting testicular sperm with different motility were still unknown.

Here, we performed this retrospective data analysis aiming to determine the AOA treatment on reproductive and neonatal outcomes of NOA couples attempting ICSI with micro-TESE sperm. Updated information of the AOA’s impact on pregnancy and newborns’ outcomes after ICSI with micro-TESE sperm with different motility will be crucial for embryologist to make proper operation and for physicians to make proper recommendations.

## 2 Materials and methods

### 2.1 Study design, setting, and participants

This retrospective study included 235 couples who visited the Reproductive Medicine Center of Third Affiliated Hospital of Guangzhou Medical University for ICSI between January 2018 and December 2020. The male partners were diagnosed with NOA, and their sperm was retrieved by micro-TESE in our hospital. Diagnosis of NOA was based on previous reports obtained at our center (17). All patients who initiated the assisted reproductive cycle had at least one or more tubes of cryopreserved testicular sperm. Testicular tissue suspension was frozen according to our center’s usual methods (18). The female patients underwent ovarian stimulation using recombinant follicle-stimulating hormone or human menopausal gonadotropin combined follicle-stimulating hormone antagonists or follicle-stimulating hormone-agonist (19). On the day of oocyte retrieval, testicular sperm was thawed, and ICSI was performed for 331 cycles. AOA was approved by the Ethics Committee of the Third Affiliated Hospital of Guangzhou Medical University and was carried out in accordance with the Helsinki Declaration. Due to the study’s retrospective nature, informed consent was not required, and patient data were used anonymously.

### 2.2 Testicular tissue suspension thawed on oocyte retrieval day

After the oocytes were retrieved, a thawing process was immediately performed. The cryovial was removed from the liquid nitrogen container and placed at 24 ± 2 °C for 10 min. Then, the thawed liquid was transferred to a fresh 15 mL centrifuge tube and 2 mL of SpermRinse washing buffer (Vitrolife, Sweden) was added to the tube dropwise and gently mixed. The mixture was centrifuged at 400 g for 10 min, and the supernatant was subsequently removed. The pellet was then resuspended with 1–2 mL of SpermRinse washing buffer. After the second wash, the sperm pellet was resuspended with 50–100 μL of G-IVF-Plus (Vitrolife, Sweden) fluid and placed in a CO_2_ incubator until ready to use.

### 2.3 ICSI procedure

On the day of oocyte retrieval, the ICSI dish was prepared according to usual laboratory procedures, and the incubator was preheated to 37 °C (without CO_2_) for ≥30 min. Before ICSI, the treated testicular sperm was added to G-MOPS-plus (Vitrolife, Sweden) droplets in the dish for incubation and a drop of sperm agonist containing 3.6 mmoL/L Pentoxifylline was added (20). Sperm were then observed under the microscope and transferred to PVP (Vitrolife, Sweden) droplets. After obtaining adequate available sperm, they were sequentially immobilized for ICSI.

During ICSI, motile sperm were first screened for injection; in case of insufficient motile sperm, immotile sperm can also be used for ICSI. If motile sperm and immotile sperm are used in the same oocyte retrieval cycle, the oocytes should be cultured separately according to the motility of injected sperm. Oocytes injected with motile sperm with severe teratospermia, with suspected low fertilization, or immotile sperm were placed into an AOA dish after ICSI. Oocytes injected with motile sperm were transferred directly into the embryo culture dish without AOA.

### 2.4 Artificial oocyte activation process

According to Nasr-Esfahani (15), after ICSI operation, oocytes were immediately transferred to G-IVF-plus medium with a final concentration of 10 μmol/L ionomycin (Sigma, USA) and placed in an incubator at 37 °C with 6% CO_2_ for 15 min. They were washed in G-IVF-plus medium for three successive rounds and then transferred to G1-plus (Vitrolife, Sweden) medium droplets that were balanced overnight in an embryo incubator for routine culture.

### 2.5 Evaluation criteria for embryo culture

According to laboratory evaluation criteria, which indicate that the standard reference of available embryos on day 3 is the number of pronucleus (PN) on day 1, the number of blastomere of embryos on day 3 was ≥5, the difference of blastomere size was ≤30%, and the proportion of fragments was ≤20%. The high-score embryos were those with 7–9 blastomeres and no difference in size. The fertilization rate is defined as the number of zygotes of all observed pronuclei divided by the number of injected oocytes on day 1. The available embryo rate is the ratio between the number of available embryos and the number of zygotes. The high-score embryo rate is the ratio between the number of high-score embryos and the number of 2PN. The no available embryo cycle rate is the ratio of the number of ICSI cycles of no available embryos to the total number of ICSI cycles. The clinical pregnancy rate is the ratio of the number of clinical pregnancy cycles to the number of total transfer cycles. Finally, the live birth rate is the number of live birth cycles divided by the total number of transfer cycles.

### 2.6 Statistical analysis

Statistical analyses were performed with SPSS software for Windows, version 26.0 (SPSS, Chicago, IL, USA). Normally-distributed quantitative parameters were expressed as mean ± standard deviation (SD) and compared using Students’ *t* test or one-way ANOVA when appropriate. Quantitative parameters which were not normally-distributed were expressed as median (25th and 75th quartiles) and compared using Mann–Whitney U test. Comparisons of frequencies and proportions were made using Chi-squared test. A *P* value <0.05 was considered to have statistical significance.

## 3 Results

### 3.1 Micro-TESE sperm morphology and AOA status of patients with NOA

**Figure 1:**
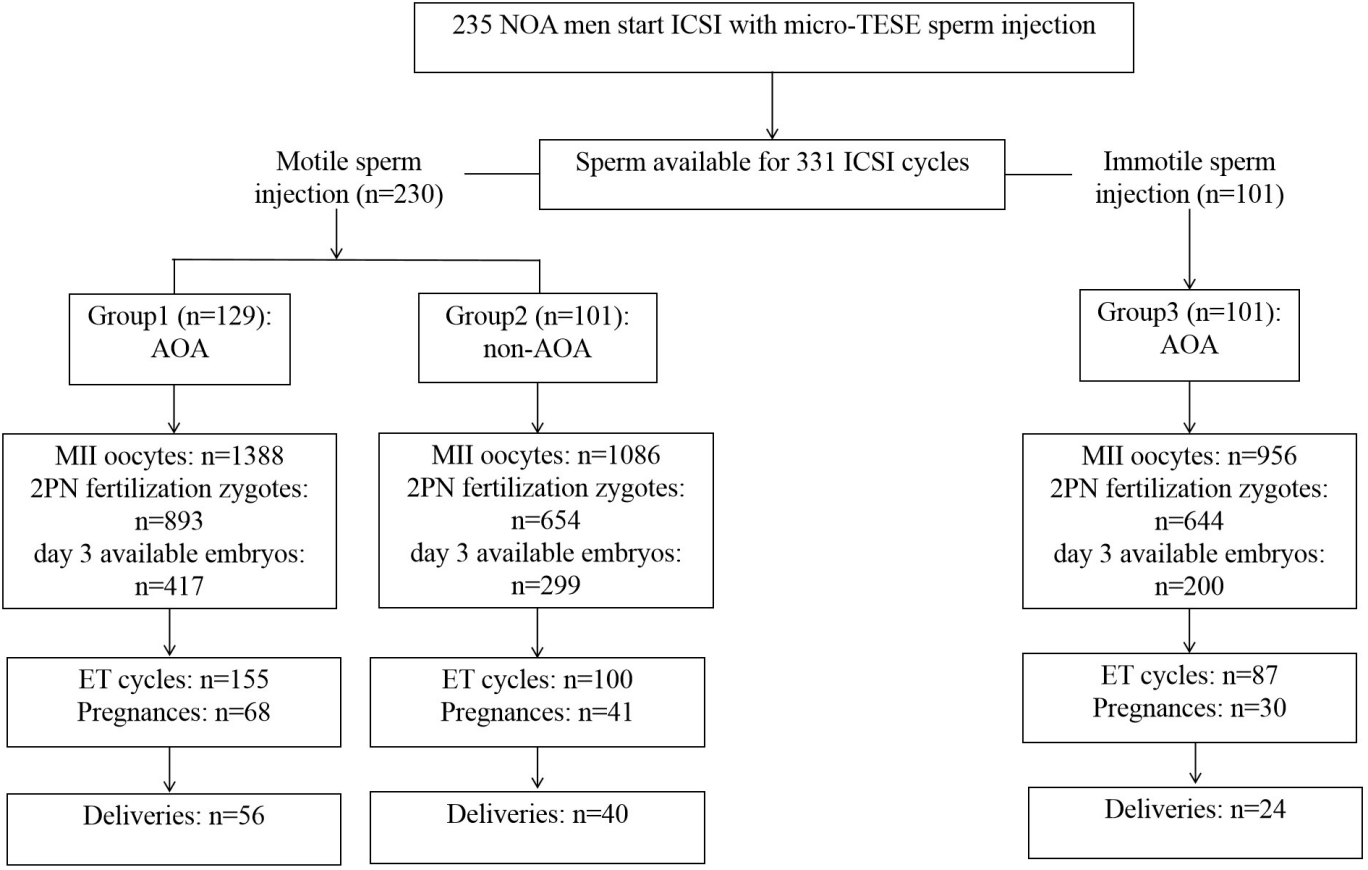
Schematic overview of the study.

**Figure 2:**
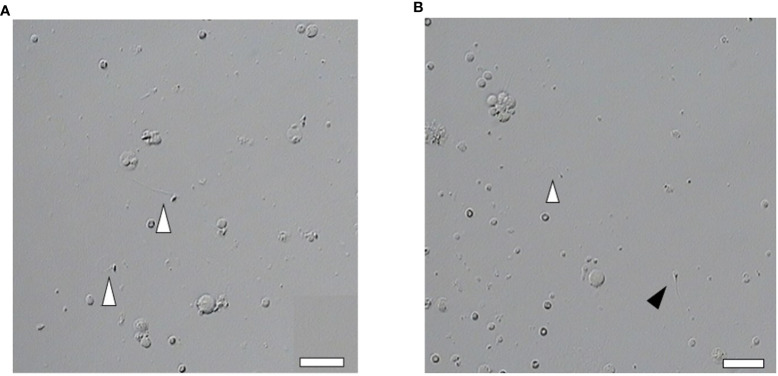
Abnormal morphology of sperm retrieved by micro-TESE. **(A, B)** Most of the sperm retrieved by micro-TESE are teratogenic, but occasionally normal sperm can be found. Open arrowhead indicates teratospermia. Solid arrowhead indicates normal morphology sperm (Scale bar, 20 μm).

A total of 331 ICSI cycles were included (**Figure 1**). The micro-TESE sperm in patients with NOA is mostly small with a pear-shaped or cone-shaped head (**Figure 2A**) under an inverted microscope. In a few samples, sperm of normal morphology can be observed (**Figure 2B**). Cycles injected with motile sperm and treated with AOA to prevent low fertilization were labeled as group 1 (129 cycles). Cycles injected with motile sperm without AOA were labeled as group 2 (101 cycles). The other 101 cycles injected with immotile sperm and treated with AOA were labeled as group 3.

### 3.2 Comparisons of basic clinical characteristics among groups

The baseline characteristics were comparable considering male age, female age, anti-Mullerian hormone (AMH), antral follicle counting (AFC), body mass index (BMI), type of infertility, and endometrial thickness among groups (**Table 1**).

**Table 1:** Baseline characteristics of the patients enrolled in this study.

	Group 1 (n=129)	Group 2 (n=101)	Group 3 (n=101)	*t/H/χ^2^/*	*P-value*
Male, Age (years)	32.89 (29.00, 35.00)	32.10 (28.00, 35.00)	31.69 (28.00, 34.00)	4.303	0.116
Female,					
Age (years)	29.96 (27.00, 32.50)	30.57 (27.00, 34.00)	29.77 (27.00, 33.00)	1.927	0.382
Infertility duration (years)	4.31 (2.00, 5.50)	4.15 (2.00, 5.00)	4.31 (2.00, 5.00)	0.699	0.705
AMH (ng/mL)	5.39 (2.28, 6.99)	5.13 (2.53, 6.30)	4.31 (2.08, 5.46)	4.264	0.119
Total AFC	20.14 (13.00, 24.00)	21.03 (13.00, 26.00)	19.84 (15.00, 24.00)	0.006	0.997
BMI (kg/m^2^)	22.42 (19.82, 23.95)	21.69 (19.20, 23.20)	21.97 (19.30, 23.81)	3.687	0.158
Type of infertility % (n)				0.097	0.953
Primary	83.72% (108)	83.17% (84)	82.18% (83)		
Secondary	16.28% (21)	16.83% (17)	17.82% (18)		
Endometrial thickness (mm)	9.91 ± 2.40	10.38 ± 2.38	10.12 ± 2.34	1.100	0.334
Endometrial type % (n)				3.274	0.195
Type A	42.64% (55)	31.68% (32)	41.58% (42)		
Type B	57.36% (74)	68.32% (69)	58.42% (59)		
Type C and others	0	0	0		

Values are mean ± standard deviation; median (Quartile1, Quartile3) or percent (n).

AMH, anti-mullerian hormone; AFC, antral follicle count; BMI, body mass index.

### 3.3 Comparison of ICSI outcomes after AOA or non-AOA; motile sperm or immotile sperm injection

As demonstrated in **Table 2**, group 1 (AOA with motile sperm injection) have comparable female age and MII number compared with group 2 (non-AOA group). In group 1, cycles had higher fertilization rate (72.77% *vs.* 67.59%, *p*=0.005) and 2PN fertilization rate (64.33% *vs.* 60.22%, *p*=0.036) compared with group 2. The 1PN fertilization rate (7.49% *vs.* 5.89%, *p*=0.117), multi-PN fertilization rate (0.94% *vs.* 1.47%, *p*=0.218), available embryo rate (41.29% *vs*. 40.74%, *p*=0.817), high-score embryo rate (13.44% *vs.* 15.44%, *p*=0.265), and no embryo for transfer cycle rate (10.85% *vs.* 9.90%, *p*=0.815) were comparable between the two groups (**Table 2**).

**Table 2:** Comparison of the ICSI outcomes between AOA and non-AOA after motile or immotile sperm injection.

	Group 1	Group 2	Group 3	*P-*value	*P1*-value	*P2*-value
NO. of ICSI cycles	129	101	101	/	/	/
Age (years)	29.96 (27, 33)	30.57 (27, 34)	29.77 (27, 33)	0.382	0.243	0.244
NO. of MII	10.85 (6, 15)	10.75 (7, 15)	9.47 (6¸12)	0.143	0.848	0.106
Fertilization rate (%)	72.77 (1 010/1 388)^a^	67.59 (734/1 086)^b^	78.56 (751/956)^c^	0.000^*^	0.005^*^	0.000^*^
2PN rate (%)	64.33 (893/1 388)^a,b^	60.22 (654/1 086)^b^	67.36 (644/956)^a^	0.003^*^	0.036^*^	0.001^*^
1PN rate (%)	7.49(104/1 388)^a^	5.89 (64/1 086)^a^	10.77 (103/956)^b^	0.000^*^	0.117	0.000^*^
3PN rate (%)	0.94 (13/1 388)^a,b^	1.47 (16/1 086)^b^	0.42 (4/956)^a^	0.051	0.218	0.016^*^
Rate of available embryos on day 3 (%)^1^	41.29 (417/1 010)^a^	40.74 (299/734)^a^	26.63 (200/751)^b^	0.000^*^	0.817	0.000^*^
Rate of high-score embryos on day 3 (%)^2^	13.44 (120/893)^a^	15.44 (101/654)^a^	6.99 (45/644)^b^	0.000^*^	0.265	0.000^*^
Rate without an embryo for transfer cycle (%)	10.85 (14/129)^a^	9.90 (10/101)^a^	23.76 (24/101)^b^	0.006^*^	0.815	0.008^*^

Values are mean ± standard deviation; median (Quartile1, Quartile3) or percent (n).

Group 1, AOA after motile sperm injection; Group 2, non-AOA after motile sperm injection; Group 3, AOA after immotile sperm injection.

P1, Group 1 compared with group 2; P2, Group 3 compared with group 2. Focusing on P1 and P2, the comparison between group 1 and group 3 was not performed.

^a,b,c^ Different superscript letters indicate statistical significance between groups(P<.05).

*Significantly different (P<.05).

^1^Computational formula: number of available embryos on day 3/number of fertilized oocytes.

^2^Computational formula: number of high-score embryos on day 3/number of 2PN.

AOA, assisted oocyte activation; ICSI, intracytoplasmic sperm injection; MII, mature oocyte; PN, primary nucleus.

When compared with group 2, group 3 (immotile sperm injection and AOA) had comparable female age and MII number. Group 3 had higher fertilization rate (78.56% *vs.* 67.59%, *p*=0.000), 2PN fertilization rate (67.36% *vs.* 60.22%, *p*=0.001), and no embryo for transfer cycle rate (23.76% vs. 9.90%, *p*=0.008). However, other outcomes, including available embryo rate (26.63% vs. 40.74%, *p*=0.000) and high-score embryo rate (6.99% vs. 15.44%, *p*=0.000) were lower relative to group 2 (**Table 2**).

### 3.4 Comparison of clinical outcomes after AOA or non-AOA; motile sperm or immotile sperm injection

As some ICSI cycles had more than one embryo transfer (ET), there were 155 ET cycles in group 1, 100 ET cycles in group 2, and 87 ET cycles in group 3. As demonstrated in **Table 3**, group 1 had comparable blastocyst-stage ET rate (24.37%, 24.84%, and 19.20%, *p*=0.464), implantation rate (34.87%, 31.85%, and 28.00% *p*=0.408), clinical pregnancy rate (43.87%, 41.00%, and 34.48%, *p*=0.360), and live birth rate (36.13%, 40.00%, and 27.59%, p=0.194) compared with group 2 and group 3, respectively. However, in group 3, more women had miscarriage compared with group 2 (20.00% *vs.* 2.44%, *p*=0.014). Group 1 had a higher rate of pregnancy with their first ET (67.09%, *p*=0.004) compared with the other groups (**Table 3**).

**Table 3:** Comparison of clinical outcomes between AOA and non-AOA after motile or immotile sperm injection.

	Group 1	Group 2	Group 3	*P-value*	*P1*-value	*P2*-value
No. of ET cycles	155	100	87		/	/
Age (years)	29.95 (27, 33)	30.25 (27, 34)	29.11 (27, 32)	0.366	0.346	0.107
Embryos per transfer	1.51 (1, 2)	1.56 (1, 2)	1.41 (1, 2)	0.131	0.433	0.057
Blastocyst-stage ET rate (%)	24.37 (58/238)	24.84 (39/157)	19.20 (24/125)	0.464	0.915	0.259
Implantation rate (%)	34.87 (83/238)	31.85 (50/157)	28.00 (35/125)	0.408	0.533	0.484
Clinical pregnancy rate (%)	43.87 (68/155)	41.00 (41/100)	34.48 (30/87)	0.360	0.651	0.360
Miscarriage rate (%)	17.65 (12/68)^a,b^	2.44 (1/41)^b^	20.00 (6/30)^a^	0.043^*^	0.018^*^	0.014^*^
Pregnancy rate of first ET (%)	67.09 (53/79)^a^	50.00 (32/64)^a,b^	40.28 (29/72)^b^	0.004^*^	0.039^*^	0.255
Live birth rate (%)	36.13 (56/155)	40.00 (40/100)	27.59 (24/87)	0.194	0.533	0.074
Neonatal outcomes of embryo transfer				0.369	0.634	0.307
Singletons (%)	78.57 (44/56)	82.50 (33/40)	91.67 (22/24)			
Twins (%)	21.43 (12/56)	17.50 (7/40)	8.33 (2/24)			
Baby’s sex				0.030^*^	0.011^*^	0.606
Birth babies	68	47	26			
Male (%)	39.71 (27/68)^a^	63.83 (30/47)^b^	57.69 (15/26)^a,b^			
Female (%)	60.29 (41/68)	36.17 (17/47)	42.31 (11/26)			
Birth weight (g)						
Singletons	3177.84 ± 435.03	3175.76 ± 444.40	3200.45 ± 424.29	0.975	0.795	0.970
Twins	2220.83 ± 646.29	2156.64 ± 671.03	2358.75 ± 130.28	0.849	0.850	0.110
Body length (cm)						
Singletons	49.34 (48.00, 50.75)	50.06 (49.00, 51.00)	50.00 (50.00, 50.25)	0.708	0.607	0.661
Twins	46.00 (45.25, 48.75)	44.29 (43.00, 48.00)	47.00 (46.00, 48.00)	0.712	0.455	0.518
Early neonatal death rate (%)	0	0	0			
Birth defect rate (%)	0	0	0			

Values are mean ± standard deviation; median (Quartile1, Quartile3) or percent (n).

Group 1, AOA after motile sperm injection; Group 2, non-AOA after motile sperm injection; Group 3, AOA after immotile sperm injection.

P1, Group 1 compared with group 2; P2, Group 3 compared with group 2. Focusing on P1 and P2, the comparison between group 1 and group 3 was not performed.

^a,b^ Different superscript letters indicate statistical significance between groups (P<.05).

*Significantly different (P<.05).

AOA, assisted oocyte activation; ET, embryo transfer.

Neonatal outcomes, including singleton and twin births rate, baby’s birth weight, and baby’s body length were comparable among the three groups. In group 2, women had more male newborn birth compared with group 1 (63.83% *vs.* 39.71%, *p*=0.011).

## 4 Discussion

The clinical indications for the use of AOA in assisted reproductive techniques mainly include complete fertilization failure or fertilization rate ≤30% in the patient’s previous ICSI (10), and severe teratospermia (such as round head sperm and acrosome defects) (13, 15). AOA methods include mechanical stimulation and chemical activation. In the mechanical stimulation method, activation of oocytes was assisted by the suction of injection pipette directly during ICSI to improve fertilization rate (5). The chemical activation method mainly uses calcium ionophore of a certain concentration (calcimycin A23187 or ionomycin) to incubate with oocytes for 10 to 15 min after ICSI (15, 21). These outcomes suggested that AOA could effectively improve ICSI fertilization rate; however, Meerschaut et al. (21) believed that not all patients with low ICSI fertilization rate could benefit from AOA.

Due to the poor spermatogenic function of patients with NOA, sperms obtained by micro-TESE often have extremely poor motility, acrosome defects, and other morphological abnormalities, that often lead to low fertilization rate, poor embryo quality, and low clinical pregnancy rate due to the inability to activate oocytes and initiate the second meiosis after ICSI (22). To improve the ICSI and clinical outcomes with severely abnormal sperm, AOA was applied on the oocytes of some patients whose partners had produced motile sperm and for partners of all patients who produced only immotile sperm after micro-TESE.

The main strength of this study is the comprehensive analysis of clinical outcomes for patients with NOA with different motile sperm retrieved by micro-TESE who underwent ICSI cycles, which is rarely reported in previous literature.

### 4.1 Motile sperm improves embryo and live birth outcomes in patients with NOA

NOA is caused by Y-chromosome microdeletions and chromosomal abnormalities, as well as non-genetic etiologies, such as cryptorchidism, heat exposure, infections, and chemoradiotherapy (23). The sperm of patients with NOA for ICSI can be retrieved through micro-TESE surgery. Usually, the selection of motile sperm is given priority, but immotile sperm with normal morphology can also be chosen when the number of motile sperm is insufficient. However, during micro-TESE surgery, testicular spermatozoa DNA fragmentation could increase significantly after cryopreservation in cryotubes, possibly due to the formation of reactive oxygen species that cause chromatin damage (24–26). To improve laboratory and clinical outcomes in this extremely abnormal sperm condition, we performed AOA treatment on the oocytes of some patients with motile or immotile sperm obtained through micro-TESE surgery after ICSI. The laboratory and clinical outcomes of ICSI treated with or without AOA were compared and analyzed.

Our results showed that after ICSI, patients with motile sperm had greater availability of embryos, high-score embryos, and lower possibility of no embryo for transfer compared with patients with immotile sperm. This suggests that patients with NOA with motile sperm are more likely to become biological fathers than those with immotile sperm. Thus, when micro-TESE sperm is needed for ICSI, efforts should be made to retrieve motile sperm.

### 4.2 Motile sperm and non-AOA treatment benefits live birth outcomes in patients with NOA

We compared the fertilization, embryo development, pregnancy outcomes, and live birth rate of AOA and non-AOA treatment in patients injected with motile sperm. AOA improved the rates of fertilization and pregnancy obtained from the first ET of patients with NOA, but miscarriage rate increased. Furthermore, our study showed that AOA treatment did not improve the quality of embryos after fertilization. This is consistent with a study reported that AOA can effectively improve fertilization from ICSI, but not all patients with low fertilization rates can benefit (21).

AOA used in this study used Ca^2+^ ion carriers to assist oocyte activation. It has been reported that specific Ca^2+^ signatures would likely impact cellular events during oocyte activation (27) and subsequent embryonic development (28). Moreover, researchers found that a physiological or artificial lack of Ca^2+^ signaling during oocyte activation in mice and humans could impair preimplantation development, blastocyst quality, and gene expression profiling (29, 30). Therefore, patients with low Ca^2+^ signaling patterns may benefit from AOA treatment for improving embryological and clinical outcomes. In our study, there were no significant differences in available embryos on day 3, high-score embryos on day 3, blastocyst-stage ET, clinical pregnancy, implantation, and live birth rates between patients with AOA and non-AOA injected with motile sperm, which indicated that NOA patients with motile sperm may have normal Ca^2+^ signaling patterns and normal embryonic development activation levels. Under these conditions, an additional AOA operation did not improve embryo quality. Conversely, the excessive artificial Ca^2+^ oscillations created by AOA might affect other downstream sequences, such as mitochondrial metabolism, or other critical developmental pathways (31, 32). Notably, current studies have focused on congenital abnormalities, birth weight, and neurodevelopmental outcomes of AOA-born children; however, long-term follow-up data were absent and need to be investigated in future studies. Therefore, we recommend that patients with NOA do not need AOA operation after ICSI using motile sperm.

### 4.3 Motile sperm is not a determinant of live birth outcomes in patients with NOA

Since AOA did not improve embryo quality after motile sperm ICSI, we analyzed whether AOA could improve live birth outcomes of immotile sperm injection, by comparing patients who retrieved motile sperm after AOA with those who retrieved motile sperm without AOA. In patients with no motile sperm, the 2PN fertilization rate was significantly higher than that of the patients receiving motile sperm injection without AOA. However, the available embryos on day 3 and the high-score embryos on day 3 rates were significantly lower than those of the patients ICSI with motile sperm. In addition, the proportion of no embryos for transfer was still high. Although the embryo quality of patients with AOA injected with immotile sperm was poor than that of patients without AOA injected with motile sperm, the rates of pregnancy and live birth was comparable between them. This is consistent with previous studies in which the clinical pregnancy rates of patients with AOA and immotile sperm injection were similar to those with motile sperm injection alone (33). Although some studies have reported that immotile sperm after thawing may still lead to normal fertilization (34, 35), ICSI with immotile sperm after thawing usually presents lower fertilization rates than when using motile sperm. Furthermore, several studies have reported that the addition of AOA with ionomycin after ICSI could significantly increase the fertilization rate in patients with severe teratozoospermia (4, 36). For example, Ebner et al. (37) showed that AOA with a Ca^2+^ ionophore could enhance fertilization in patients with cryptozoospermia. In our study, we applied AOA to some patients with NOA using thawed motile or immotile micro-TESE sperm, which significantly improved fertilization rate and live birth rate, regardless of sperm motility status. Considering the rarity of sperm in patients with NOA, the uncertainty of the survival status of immotile sperm, and the acceptable live birth rate of immotile sperm brought by AOA, it is recommended that patients without motile sperm use immotile sperm for ICSI, followed by AOA with a Ca^2+^ ionophore.

### 4.4 Patients with NOA have higher miscarriage rate after AOA

In this study, the miscarriage rates in patients with AOA with both motile and immotile sperm were significantly higher compared with patients without AOA, and higher than the overall ART spontaneous abortions (10–15%) reported in China (38). The high miscarriage rate was probably related to the fact AOA operation cannot improve embryo quality. Furthermore, the motile micro-TESE sperm injected can be fertilized normally without AOA, indicating the motile micro-TESE sperm may have normal Ca^2+^ signaling patterns and normal activation levels for fertilization and subsequent embryonic development. Under this condition, an additional AOA operation did not improve the embryo quality. Conversely, the excessive artificial Ca^2+^ oscillations created by AOA might affect other critical developmental pathways. The high miscarriage rate of immotile sperm injection may be related to the poor sperm quality in patients with NOA. These patients retrieved sperm through micro-TESE with poor morphology and motility, increased oxidative stress levels, and higher DNA fragmentation degrees. In addition, after cryopreservation, the formation of reactive oxygen species significantly increases DNA fragmentation in testicular sperm, resulting in chromatin damage, aneuploidy, mosaicism, and DNA damage that contribute to poor embryo quality and increased miscarriage rates (32, 33).

### 4.5 Limitations

This study has some limitations, including the small sample size of the group of embryo transfer patients injected with immotile sperm, the need for more follow-up data on live births, and patient selection bias. In future, further focus should be given to congenital abnormalities, birth weight, and neurodevelopmental outcomes of AOA-born children, and long-term follow-up data need to be collected.

## 5 Conclusions

In summary, patients with NOA do not need AOA after injection of motile micro-TESE sperm; the fertilization rate of 2PN can reach 60.22%, while live birth rates can reach 40%. It is suggested that immotile sperm with normal morphology is used for ICSI in patients without motile sperm, who then undergo AOA; in this way, the live birth rate of immotile sperm injection can reach 27.59%, which is not significantly different from that of motile sperm injection, and the 2PN fertilization rate can reach 67.36%. It should be noted that there was a significant increase in miscarriage rates after AOA with both motile and immotile sperm.

## Data availability statement

The raw data supporting the conclusions of this article will be made available by the authors, without undue reservation.

## Ethics statement

The studies involving human participants were reviewed and approved by The Ethics Committee of the Third Affiliated Hospital of Guangzhou Medical University. Written informed consent for participation was not required for this study in accordance with the national legislation and the institutional requirements.

## Author contributions

The contributions of all authors were as follows: conceptualization and data curation: LeL and HD Writing-original draft: XZ. Statistical analysis: LiL. Data collection: WZ, YL, and YM. All authors contributed to the article and approved the submitted version.
